# Versican is differentially regulated in the adventitial and medial layers of human vein grafts

**DOI:** 10.1371/journal.pone.0204045

**Published:** 2018-09-28

**Authors:** Richard D. Kenagy, Shinsuke Kikuchi, Steve P. Evanko, Matthijs S. Ruiter, Marco Piola, Alban Longchamp, Maurizio Pesce, Monica Soncini, Sébastien Deglise, Gianfranco B. Fiore, Jacques-Antoine Haefliger, Tannin A. Schmidt, Mark W. Majesky, Michael Sobel, Thomas N. Wight

**Affiliations:** 1 Center for Cardiovascular Biology, Institute for Stem Cells and Regenerative Medicine, and Department of Surgery, University of Washington, Seattle, WA, United States of America; 2 Department of Vascular Surgery, Asahikawa Medical University, Asahikawa, Japan; 3 Matrix Biology Program, Benaroya Research Institute, Seattle, WA, United States of America; 4 Cardiovascular Tissue Engineering Unit—Centro Cardiologico Monzino, IRCCS, Via Parea, 4, Milan, Italy; 5 Dipartimento di Elettronica, Informazione e Bioingegneria, Politecnico di Milano, Milan, Italy; 6 Department of Vascular Surgery, CHUV | Lausanne University Hospital, Lausanne, Switzerland; 7 Biomedical Engineering Department, School of Dental Medicine, University of Connecticut Health Center, Farmington, CT, United States of America; 8 Center for Developmental Biology and Regenerative Medicine, Seattle Children's Research Institute, Seattle, WA, United States of America; 9 Division of Vascular Surgery, VA Puget Sound Health Care System, University of Washington, Seattle, WA, United States of America; University of Patras, GREECE

## Abstract

Changes in extracellular matrix proteins may contribute significantly to the adaptation of vein grafts to the arterial circulation. We examined the production and distribution of versican and hyaluronan in intact human vein rings cultured ex vivo, veins perfused ex vivo, and cultured venous adventitial and smooth muscle cells. Immunohistochemistry revealed higher levels of versican in the intima/media compared to the adventitia, and no differences in hyaluronan. In the vasa vasorum, versican and hyaluronan associated with CD34^+^ progenitor cells. Culturing the vein rings for 14 days revealed increased versican immunostaining of 30–40% in all layers, with no changes in hyaluronan. Changes in versican accumulation appear to result from increased synthesis in the intima/media and decreased degradation in the adventitia as versican transcripts were increased in the intima/media, but unchanged in the adventitia, and versikine (the ADAMTS-mediated cleavage product of versican) was increased in the intima/media, but decreased in the adventitia. In perfused human veins, versican was specifically increased in the intima/media in the presence of venous pressure, but not with arterial pressure. Unexpectedly, cultured adventitial cells express and accumulate more versican and hyaluronan than smooth muscle cells. These data demonstrate a differential regulation of versican and hyaluronan in human venous adventitia vs. intima/media and suggest distinct functions for these extracellular matrix macromolecules in these venous wall compartments during the adaptive response of vein grafts to the arterial circulation.

## Introduction

Saphenous veins continue to be used to bypass advanced arterial atherosclerotic lesions of the heart and limbs. However, severe luminal narrowing, a primary cause of failure, develops during the first 1–2 years in ~30% of vein grafts due to pathological remodeling and intimal hyperplasia. While there are also early failures (< 1 month) mainly due to surgical technique, and very late failures (>5 years) due to the progression of native atherosclerosis, stenoses and narrowing of the vein continue to be the main limiting factor for bypass success [1, 2].

In human veins, intimal lesions contain mesenchymal cells with large amounts of extracellular matrix (ECM) rich in versican and hyaluronan [3, 4]. Animal and human vein grafts show a rapid loss of cells in the media after graft implantation due to cell death. Based on animal models, this is followed by thickening of the intimal and medial layers as a consequence of cell migration, cell proliferation, and deposition of ECM. However, the origins of the cells that form the hyperplastic intima and the cellular source of the ECM are uncertain. Animal models have also shown that the cells involved in this response include medial smooth muscle cells (SMCs), progenitor cells from the blood, and adventitial cells [1, 2]. Since versican, versikine (the ADAMTS-mediated cleavage product of versican), and hyaluronan are known to be involved in cell proliferation, cell migration, and intimal hyperplasia[5, 6], we evaluated the ability of both SMCs and adventitial cells to synthesize, deposit, and degrade versican and hyaluronan given the evidence from animal models that both types of cells contribute to neointimal hyperplasia [7, 8]. Furthermore, we examined the pattern of versican and hylauronan accumulation in two models of the intimal hyperplastic response: ex vivo cultures of veins and a flow model of arterial or venous pressure. These experiments focus on further defining the involvement of two specific ECM components, hyaluronan and versican, in events associated with human saphenous graft failure.

## Methods

### Vein rings, tissue culture, and cell culture

Human saphenous vein remnants were obtained anonymously from patients undergoing coronary artery bypass or peripheral vascular bypass operations under protocols approved by the Institutional Review Boards of the University of Washington or the Benaroya Research Institute at Virginia Mason. All vein specimens were kept in buffered saline during surgery (shown to be the best solution [9]). After the surgeon had identified the remnant specimen, it was placed into buffered Dulbecco’s Modified Eagle’s medium (DMEM; 10 mM Hepes, pH 7.4), while still in the operating room. Veins were maintained at room temperature during prompt transit to the laboratory and processing began within 2 hours.

For tissue culture (coronary artery bypass and peripheral vascular bypass veins) or for obtaining cells for culture (only peripheral vascular bypass veins), veins were dissected free of loose, extraneous tissue and the veins were cut into rings for culture in 20% fetal bovine serum (FBS). These intact rings were subsequently cultured ex vivo (floating vein rings). Other segments (that were free of valves or intraoperative blue dye markings) were opened longitudinally, and the endothelium removed by gently wiping with a cotton-tipped swab. The intimal/medial layer was dissected from the adventitial layer in a natural plane (see this published reference for an image of this dissection[10]). For tissue culture, these separate outer and inner layers of vein (~ 1 cm^2^/condition) were maintained in 20% FBS/DMEM for the indicated time. To obtain cultured cells, 2.5 mm^2^ explants of the outer and inner wall layers were made using a McIlwain tissue chopper (customized for up to 4 mm^2^). Explants were maintained in 20% FBS/DMEM. When cells around the explants became confluent (2–3 weeks), medium was changed to Smooth Muscle Cell Growth Medium (Cell Applications, Inc.), which contains 5% FBS and undisclosed amounts of EGF, FGF2, insulin, and heparin (personal communication, James Yu, Cell Applications). This medium was also used for subsequent growth of passaged cells on collagen-coated plastic ware (10 μg/ml bovine skin collagen in phosphate-buffered saline overnight at 4°C). Adventitial cells have been characterized by lack of expression of smooth muscle α-actin (SMA) in contrast to the SMA-expressing SMCs[10].

### Ex-vivo vein perfusion

Veins for ex vivo perfusion under venous or arterial conditions were obtained from patients undergoing coronary artery bypass operations under protocols approved by the Ethical Committees of the Centro Cardiologico Monzino (Italy) and the University of Lausanne (Switzerland). Four different modes of perfusion were utilized as described[11–14] (Table 1).

**Table 1:** Ex vivo vein perfusion models.

Ex Vivo Perfusion Model	Pressure	Flow	Oxygen	Reference
Model #1	Cycles of 10' pulsatile pressure (0.5 Hz, 80–120 mm Hg) then 2' ≤ 2 mm Hg	Cycles of 10' of no flow then 2' of 1 ml/min	20%	10, 12
Model #2	5 mm Hg	5 ml/min	20%	10, 11
Model #3	5 mm Hg	5 ml/min	20% lumenal & 5% adventitial	11
Model #4	pulsatile pressure (1.0 Hz, 90–120 mm Hg)	160 ml/min	20%	13

### ECM gene induction experiments

Here we studied gene induction of pairs of adventitial cells and intimal/medial SMCs that were grown from the dissected adventitial and medial layers of veins from peripheral bypass patients (always the same passage of 6 or less). Cells were seeded at 200,000/well in 6-well plates in 5% FBS (in Bovine Smooth Muscle Cell Basal Medium; Cell Applications, Inc.). The next day, medium was changed to serum-free medium. After 2 days, medium was changed to fresh serum-free medium. After a total of 3 days in serum-free medium, the medium was changed to serum-free medium ± 10 ng/ml PDGF-BB or 10% FBS (in two separate studies). Total RNA was isolated from cells at the times indicated using the Quick RNA mini prep kit from Zymo Research. The OD_260/280_ was always greater than 1.9 and most RIN numbers after Bioanalyzer analysis were >9.0 and all were > 8.0.

Dissected intima/media and adventitia tissue specimens (~ 1 cm^2^) were immediately placed in RNAlater at 4° C overnight and then frozen at -80° until processed. Other samples were maintained in 20% FBS/DMEM for the indicated time before placing in RNAlater. RNA was purified using Trizol extraction coupled with Quick-RNA™ MiniPrep columns as instructed with DNase I treatment (Zymo Research, Inc). The OD_260/280_ was 2.05 ± 0.02 (mean ± SEM). RIN numbers ranged from 6.5–9.5 with a mean value of 8.0 ± 0.2.

### Quantitative RT-PCR

All reagents were supplied by Life Technologies (Grand Island, NY) except as noted. Total RNA was reverse transcribed using the High Capacity Reverse Transcription cDNA Kit according to the manufacturer’s instructions. qRT-PCR was performed using the ABI 7900HT Fast Real-Time PCR System. HAS1-3, and versican gene expression was determined using Taqman Gene Expression AssaysHs00758053_m1, Hs00193435_m1, Hs00193436_m1, and Hs00171642_m1, respectively. One pooled RNA preparation from human saphenous vein adventitial cells was used for standards and primer efficiencies averaged from 90% to 100%. Results were normalized to 18S (no. 4333760) after testing RPL13, RPL27, GUSB, HMBS, HPRT, TBP, B2M, SDHA, UBC and 18S using Bestkeeper (http://www.wzw.tum.de/gene-quantification/bestkeeper.html), geNorm (http://medgen.ugent.be/wjvdesomp/genorm/), and Normfinder (http://moma.dk/normfinder-software).

### RNA purification and RNA-Seq

Dissected intima/media and adventitia specimens (~ 1 cm^2^/condition) were immediately placed in RNAlater at 4° overnight and then frozen at -80° until processed. Other samples were maintained in 20% FBS/DMEM for 2 days before placing in RNAlater. RNA was purified using Trizol extraction coupled with Quick-RNA™ MiniPrep columns as instructed with DNase I treatment (Zymo Research, Inc). The OD260/OD280 was 2.05 ± 0.02 (Mean ± SEM, N = 20). Bioanalyzer RIN numbers were 8.04 ± 0.22 (mean ± SEM, N = 20) and ranged from 6.5 to 9.5. Sequencing libraries were constructed from total RNA using Kapa Stranded RNA-Seq Kit with RiboErase(HMR). Paired-end 76 nt sequencing was carried out using Illumina NextSeq 500 with samples having 60–122 million reads. Base-calling software was CASAVA(version 1.8.2). Data pre-processing was done using the tool FastQC. The reads were aligned to the GRCh38 transcriptome using the salmon aligner[15]. Alignments were to the transcriptome (e.g., the set of known transcripts for GRCh38, from Ensembl). The counts for each transcript were then collapsed to counts/gene using the Bioconductor tximport package[16]. Comparisons were made using Bioconductor edgeR package[17]. The data from this study will be published as a whole and made public in another paper that is in preparation.

### Histochemistry/Immunochemistry

Rings of saphenous veins were fixed in 10% formalin for 24 h at 4° C, embedded in paraffin, and sectioned for histochemical and immunohistochemical staining. Movat’s was performed as described[18]. All steps for immunohistochemistry were performed on a Leica Bond (Leica Microsystems Inc, Buffalo Grove, IL) using the Leica Bond Polymer Detection Kit (Leica Microsystems # DS9800). This detection kit contains a peroxidase block, rabbit anti-mouse post primary reagent for use with mouse primaries, a ready to use secondary goat anti-rabbit conjugated to polymeric HRP, DAB chromogen and hematoxylin counterstain. See Table 2 for information on all antibodies used. Specifically for versikine (DPEAAE, versican neoepitope[19]), sections were pretreated using heat mediated antigen retrieval with EDTA at high pH (Bond epitope retrieval solution 2). The sections were then incubated 1 h with 1.0 μg/ml rabbit anti V0, V1 Neo Antibody (Thermo Scientific #PAI-1748A) in Bond antibody diluent followed by detection. Versican immunohistochemistry required pre-treatment with 0.2U/ml chondroitinase ABC (Sigma #C3667) in 18 mM Tris, 1mM sodium acetate, 1 mg/ml BSA pH 8.0 for 1 h at 37˚C. The sections were then incubated for 1 h with 0.06μg/ml anti Versican clone 2B1 (Seikagaku# 270428) in Bond antibody diluent and detection was performed using the Bond polymer Refine detection Kit. For the versican/CD34 double stains, the Mach 4 mouse probe and Mach 4 HRP polymer were used for the anti-versican primary and the Mach 2 double stain 1 probe was used for the anti-CD34 primary (Biocare Medical, Concord MA). For the SMA (rabbit polyclonal)/CD34 double stain, the Mach 2 double stain 1 system was used. Deep Space Black (BioCare) and AbCam Stay Red were used for the HRP and AP chromogens, respectively. For hyaluronan affinity histochemistry (AFC) the Bond Intense R Detection kit, a streptavidin-horse radish peroxidase system, (Leica Microsystems, Inc.) was used with 1 μg/ml biotinylated-HABP in 0.1% BSA-PBS. Negative controls were Chrompure mouse or rabbit IgGs (Jackson ImmunoResearch Laboratories) in Bond antibody diluent. Representative negative IgG controls, which showed no staining, are shown along with matching positives in S1 Fig. Hyaluronidase treatment removed HABP staining demonstrating specificity of the HABP staining (S1 Fig).

**Table 2:** Antibodies and reagents used in this study.

Antigen	Antibody/Reagent	Species	Concentration
Versican	2B1 (270428 Seikagaku)	mouse	0.06 μg/ml
Hyaluronan	biotinylated-HABP (hyaluronan binding protein)	-	0.05 μg/ml
DPE Versican Neo-epitope	V0, V1 Neo Antibody (PAI-1748A Thermo Scientific)	rabbit	1.0 μg/ml
ADAMTS4	PA1-1749A Thermo Scientific	rabbit	3.75 μg/ml
ADAMTS5	PA1-1751A Affinity Bioreagents	rabbit	3.2 μg/ml
Smooth muscle alpha-actin (SMA)	ab5694 Abcam	rabbit	0.4 μg/ml
Smooth muscle alpha-actin (SMA)	1A4 (A7607 Sigma)	mouse	0.05 μg/ml
CD31	JC70A (MO823 Dako)	mouse	5.0 μg/ml
CD34	QBEND 10 (M7165 Dako)	mouse	0.15 μg/ml
negative control	Chrompure IgG (011-000-003 Jackson)	rabbit	as appropriate
negative control	Chrompure IgG (015-000-003 Jackson)	mouse	as appropriate

Versican-, HABP-, versikine-, ADAMTS4-, and ADAMTS-5-stained slides were scanned in brightfield with a 20X objective using the NanoZoomer Digital Pathology System (Hamamatsu City, Japan). The digital images were then imported into the Visiopharm quantitative digital pathology platform (Hoersholm, Denmark) for analysis of both area and intensity of staining. Using the Visiopharm Image Analysis module, three regions of interest were drawn: around the outer border of the vessel (for each vein there were duplicate sections for day 0 and for day 14), around the outer edge of the circular SMC layer, and around the lumen (the actual edges of the outer adventitia and the lumen were determined by the software). Because of the difficulty in clearly distinguishing the intima-media boundary in these specimens, we quantified the intima and media together to compare to the adventitia. Movat’s stains on different sections were used to help with drawing the adventitial/medial boundary. The software was then programed to detect positive staining for versican (or HABP etc.) and for hematoxylin using a project-specific configuration based on a threshold of pixel values. The images were processed in batch mode using this configuration to generate the desired per area outputs. The feature band HDAB (hemotoxylin/diaminobenzidine)–DAB (diaminobenzidine) was used to calculate the mean intensity (the inverse was used with black = 256 and white = 0) of versican within the regions of interest. Luminal areas and areas within the adventitial/medial boundary (AMB; used as a measure of vessel remodeling) were determined on Movat’s stained sections using ImageJ (1.37v). Loss of lumen area was defined as (1-(lumen area day 14/lumen area day 0))*100) and vein contraction was defined as (1-(AMB day 14/AMB day 0))*100).

### Immunocytochemistry

Versican was localized using monoclonal antibody 2-B-1 (Seikagaku, 2 μg/ml) or a rabbit anti-versican (Abcam ab177480, 2 μg/ml). Smooth muscle actin (SMA) was localized with a mouse anti-human SMA antibody (Dako, clone 1A4, 2 μg/ml) or a rabbit anti-SMA antibody (Sigma, 2 μg/ml). Adventitial cells and SMC on 22 mm coverslips were rinsed 3 times in PBS, fixed in neutral buffered formalin, rinsed 3 times in PBS, then permeabilized for 10 min in PBS, 0.1% Triton X-100. Primary antibody was applied in PBS containing 2% bovine serum albumin for 1 h at 22 ⁰C. Following three rinses, cells were incubated with secondary antibodies (AF488 or AF555 conjugated donkey anti-rabbit or donkey anti-mouse antibodies, 1:500, Life Technologies) in PBS 2% BSA for 1 h. After rinsing, cells were mounted with Fluoro-Gel (Electron Microscopy Sciences) containing 1 μg/ml DAPI to stain nuclei, and examined using a Leica DMIRB microscope under epifluroescence optics using a 20x objective. Images were acquired using a Spot® cooled CCD camera and imaging program. The proportion of versican and smooth muscle actin positive cells were tallied from at least 5 micrographs of randomly selected felds per cell isolate, counting a minimum of 150 cells.

### Hyaluronan enzyme linked sorbent assay (ELSA)

A modification[20] of a previously described[21] competitive ELSA in which the samples to be assayed were first mixed with bPG (the biotinylated N-terminal hyaluronan binding region of aggrecan) and then added to a hyaluronan-coated microtiter plate was used. The final signal is inversely proportional to the level of hyaluronan added to the bPG. For this assay, media and cell layers were digested with 300 μg/ml pronase in 0.5M Tris, pH 6.5 for 18 h at 37°C. Following digestion, the pronase was inactivated by heating to 100°C for 20 min.

### Western analysis

Samples of adventitial and of intimal/medial tissue were cut into small pieces and extracted by vortexing overnight at 4° C in 4M guanidine HCL. The extract was concentrated and desalted to phosphate-buffered saline by using 30kDa centrifugal filter units (Amicon Ultra). Protein content of cultured cell layers and tissue extracts was quantified using the Coomassie Protein Assay kit (Pierce, Rockford, IL). For cultured cell extracts, equal amounts of protein or volumes of medium (normalized by equal cell protein) were subjected to DEAE Sephacel chromatography. Equal volumes of isolated proteoglycans were ethanol precipitated, digested with chondroitin ABC lyase, and electrophoresed on 4–12% gradient SDS-PAGE gels. For tissue extracts, equal amounts of total protein were loaded onto the SDS-PAGE gels. Proteins were transferred to nitrocellulose and probed with anti-human versican (0.1 μg/ml AF3054 [R&D systems, Minneapolis, MN] or 0.06 μg/ml 2B1 [270428 Seikagaku] for cultured cells and tissue, respectively). Anti-actin was used as a loading control (A2103, Sigma). Results were visualized using LI-COR Odyssey® scanner and software (LI-COR Biotechnology, Lincoln, NE)[22]. Quantification of western blots (V0 plus V1 vs all proteins between 75 kD and 250 kD) was done using ImageJ software (v1.52a).

### Statistical analysis

The Wilcoxon signed rank test, unpaired t-test, or the repeated measures ANOVA were performed as appropriate using Prism 6 or SPSS (v19).

## Results

### Versican is enriched in the intima/media of fresh veins and increases in both intima/media and adventitia after vein culture

We measured the density and distribution of versican in freshly harvested veins from patients undergoing coronary or peripheral artery bypass operations, and in paired samples cultured ex vivo for 14 days. This model of floating vein rings has been validated as a model of intimal hyperplasia [23, 24].

**Fig 1:**
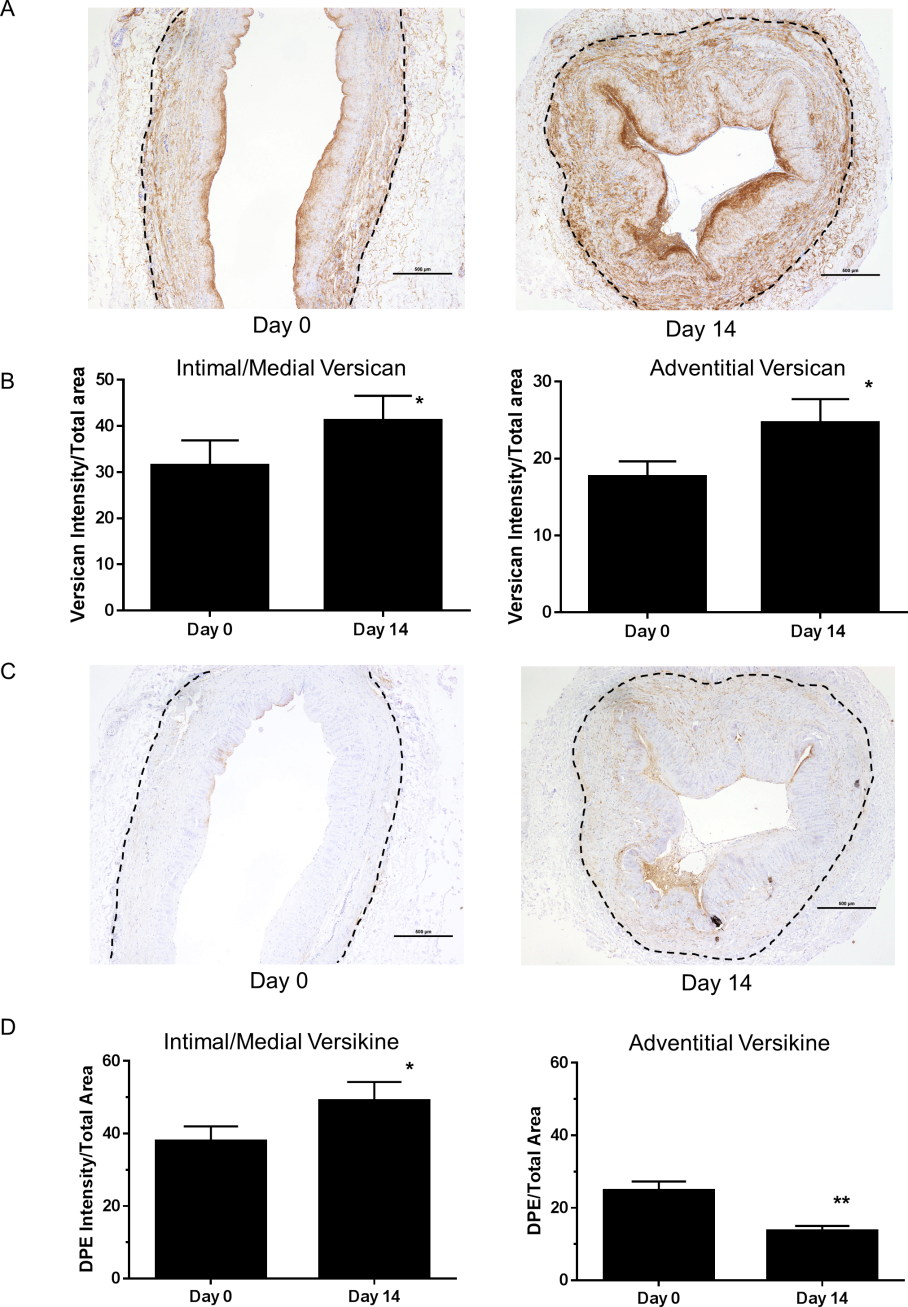
Versican and versikine immunostaining in vein rings. (A) Versican immunostaining in pre-culture and 14 day cultured floating vein rings was quantified for the intima/media and adventitia (B). The dotted line indicates the adventitial/medial boundary (* P = 0.01 day 0 vs day 14; N = 19 veins). (C) Versikine immunostaining in pre-culture and 14 day cultured floating vein rings was quantified (D) for the intima/media and adventitia (*P<0.04; **P<0.0001 day 0 vs day 14; N = 19 veins). Scale bars indicate 500 μm.

Despite significant inter-individual variation, as illustrated in S2 Fig, the staining of versican in pre-implantation vein grafts was consistently more intense in the intima (the neo-intima will be discussed below; non-immune IgG had no staining [S1 Fig]). The intima/media expressed about two-fold more versican as observed by immunostaining intensity compared to adventitia (P<0.002, day 0 intima/media vs. adventitia in Fig 1A and 1B). After 14 days of culture, there was a 31% increase in staining for versican in the intima/media and a 40% increase in the adventitia (Fig 1B). Adventitial versican co-localized with the vasa vasorum, the longitudinal cords of smooth muscle that are found in the venous adventitia, and along elastic fibers. The black elastic fibers seen in Movat’s stained sections are most prominent in the adventitia with much smaller elastic fibers in the media and internal elastic lamina (S2 Fig). In addition, there was a clear vessel contraction and loss of lumen area after 14 days (24 ± 6% and 76 ± 3%, respectively; mean ± SEM, N = 20 veins).

**Fig 2:**
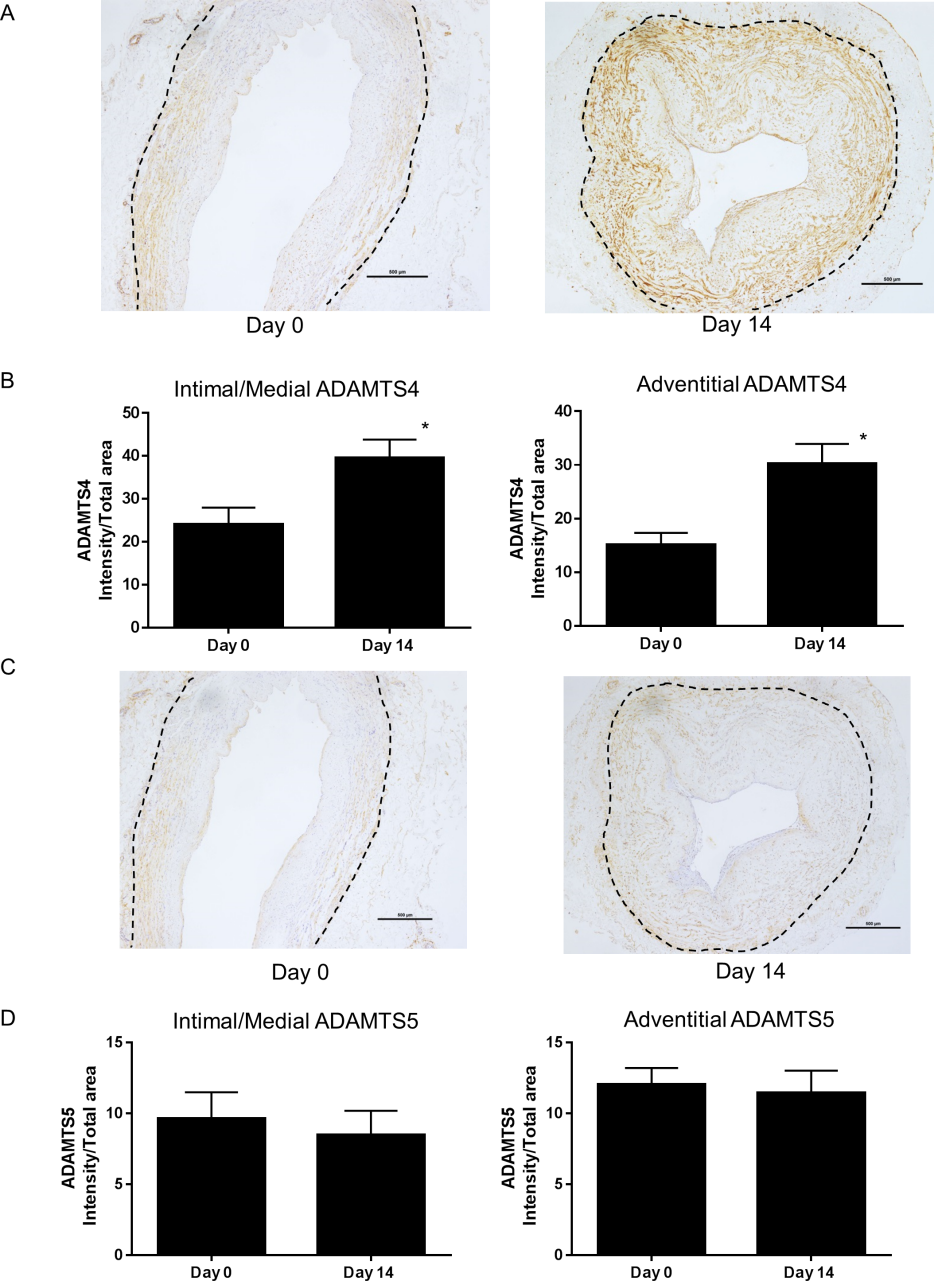
ADAMTS4 and 5 immunostaining in vein rings. (A) ADAMTS4 immunostaining in pre- and 14 day cultured floating vein rings was quantified (B) for the intima/media and adventitia (* P<0.0001 day 0 vs day 14; N = 20 veins). (C) ADAMTS5 immunostaining in pre- and 14 day cultured floating vein rings was quantified (D) for the intima/media and adventitia (N = 20 veins). The dotted line indicates the adventitial/medial boundary. Scale bars indicate 500 μm.

Levels of versican reflect the balance of synthesis and degradation; therefore, we further investigated the levels of the ADAMTS-mediated degradation product of versican, named versikine [19, 25]. Versikine levels in the pre-implantation vein graft was higher in the intima/media (P<0.002, N = 20; day 0 intima/media vs adventitia in Fig 1C and 1D; non-immune IgG had no staining [S1 Fig]) as was observed with versican. After culture for 14 days, versikine staining increased in the intima/media, but decreased in the adventitia despite levels of versican increasing in both layers (Fig 1C and 1D). Immunostaining for ADAMTS4 and 5, which are two versican-degrading ADAMTS proteinases [19, 26], was localized to the intima/media and to the vasa vasorum (Fig 2A–2D; non-immune IgG1 had no staining [S1 Fig]). ADAMTS4 increased by ~60% at day 14 in the intima/media (Fig 2A), which is consistent with increased levels of versikine. Because adventitial versikine levels were decreased, we expected that ADAMTS levels would also decrease. However, adventitial ADAMTS4 staining increased by ~90% after 14 days of culture (Fig 2B). In contrast, staining for ADAMTS5 was not changed during culture of veins (Fig 2D).

**Fig 3:**
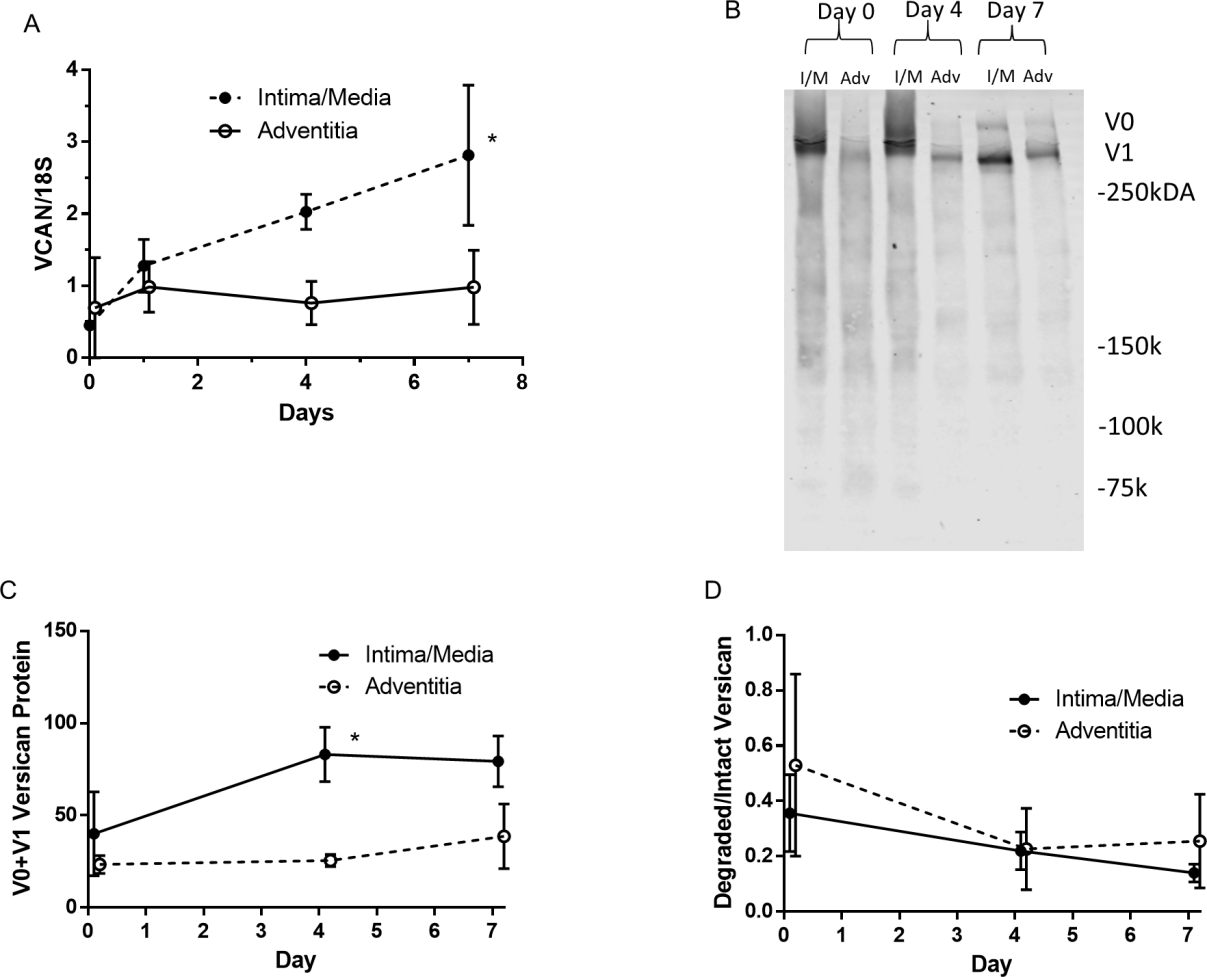
Versican induction in intimal/medial vs. adventitial tissue. Induction of versican message and protein in floating intimal/medial and adventitial tissue. (A) Versican mRNA (*P<0.02 intima/media vs. adventitia. N = 3 veins day 0, N = 6 veins days 1–7). (B) Versican western blot of tissue from one vein (“int/med” intima/media and “adv” adventitia. (C) Quantification of V0/V1 versican protein (*P<0.02 intima/media vs. adventitia mean gray value. N = 3 veins). (D) Quantification of versican degradation products (75–250 kD) relative to intact V0/V1 versican (N = 3 veins).

To address these discrepant results between changes in versican, versikine, and ADAMTS4 in the adventitia, the separated adventitia and intima/media were cultured to determine the induction of versican mRNA in these tissues. There was an induction of versican transcripts in the intimal/medial tissue over 7 days (Fig 3A, *P<0.02), but there was no significant induction in the adventitial tissue (P>.8; Fig 3A). Western blots of tissue extracts also demonstrated that levels of the V0/V1 isoforms increased in the intima/media, but not the adventitia (Fig 3B and 3C, *P<0.02). These results, combined with the decreased adventitial versikine staining suggests that the observed increase in adventitial versican during culture results from decreased versican degradation, despite the increase in ADAMTS4. This is supported by western blots, which showed decreasing amounts of versican fragments relative to intact V0/V1 with increasing time in culture for both adventitia and intima/media (Fig 3D; P<0.04 and P>0.5 for tissue or interaction by repeated measures ANOVA). In addition, RNA-Seq analysis of cultured intima/media vs. adventitia showed that TIMP3 and four serine protease inhibitors (SERPINE2, SERPINA5, SERPINI1, and SERPINF2) were more highly expressed in the adventitia compared to the intima/media (Table 3). TIMP3 is a prime inhibitor of the versican-degrading ADAMTSs and matrix metalloproteinases and these SERPINs inhibit plasmin, which also degrades versican [27].

**Table 3:** Expression of inhibitors of versican-degrading proteases in cultured adventitial and intimal/medial tissue.

	Day 0		Day 2	
	Adventitia	Int/Media	Adventitia	Int/Media
TIMP3*	2138 ± 188	1050 ± 148	749 ± 135	446 ± 20
SERPINE2**	44 ± 9	25 ± 6	2085 ± 624	682 ± 93
SERPINA5**	8.3 ± 1.3	1.8 ±0.5	2.3 ± 0.5	0.8 ± 0.1
SERPINI1**	13 ± 1	8 ±1	17 ± 3	7 ± 1
SERPINF2**	4.6 ± 1.6	0.8 ± 0.1	1.1 ± 0.2	0.4 ± 0.1

Gene expression from RNA-Seq analysis is expressed as normal numbers from paired tissues of 5 veins.

* FDR = 0.07

** FDR<0.004 adventitia vs intima/media.

### The hyaluronan staining pattern differs from that of versican

**Fig 4:**
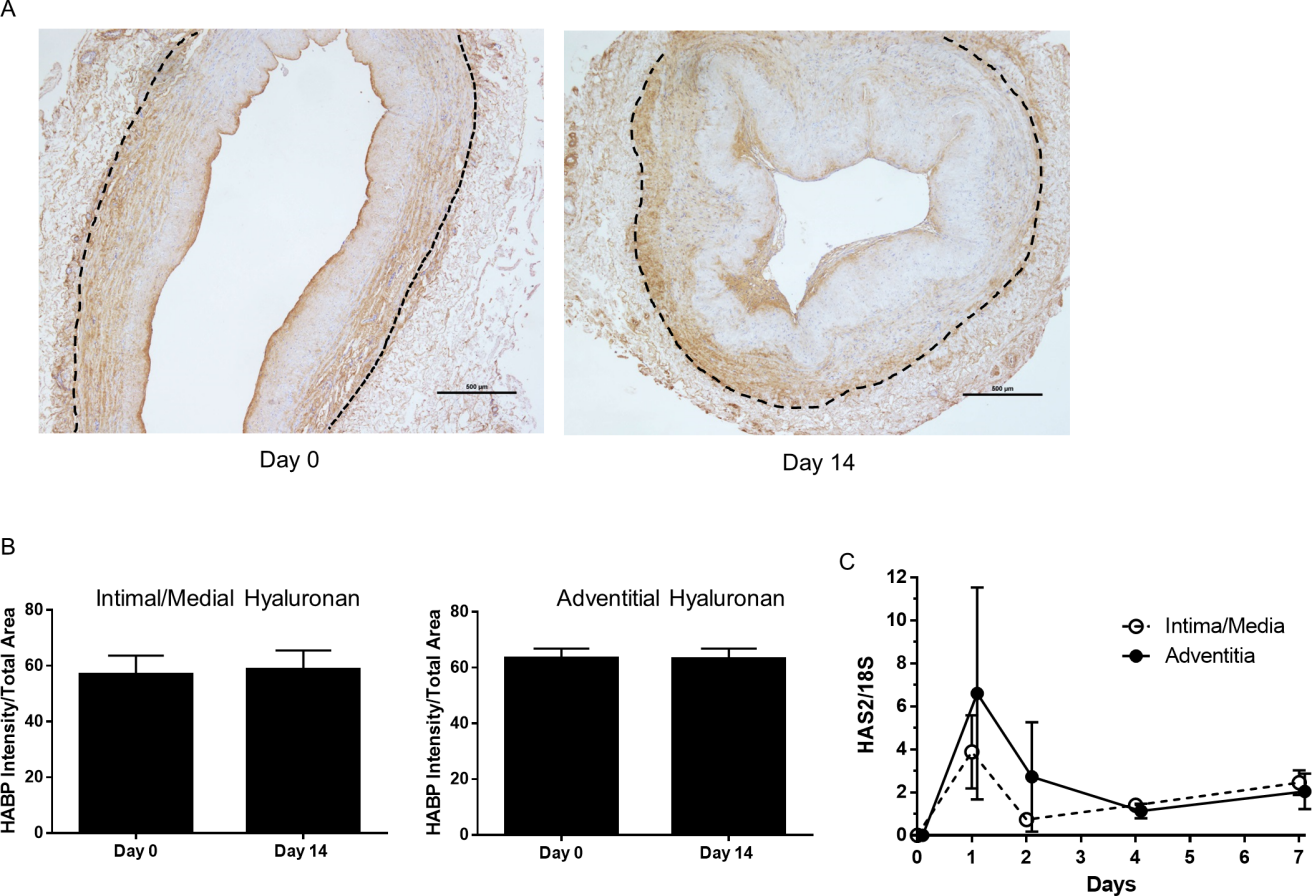
Hyaluronan staining in vein rings. (A) Hyaluronan (HABP) staining in pre-culture and 14-day cultured floating vein rings. Dotted line indicates the adventitial/medial boundary. Scale bars indicate 500 μm. (B) HABP quantification for the intima/media and adventitia. N = 19 veins. (C) Induction of HAS2 message in cultured intimal/medial and adventitial tissue. N = 3 veins day 0, N = 6 veins days 1–7.

Unlike versican, there was no difference between staining for hyaluronan in the intima/media vs the adventitia and staining did not change during the 14 day culture period (Fig 4A and 4B). When the adventitial and intima/media layers were separately cultured, we observed a transient induction of HAS2 in both adventitial and intimal/medial tissue at day 1 with no difference between the two tissues (Fig 4C). There was very low expression and absence of induction of HAS1 and HAS3 mRNA in adventitial and intimal/medial tissue (data not presented).

### Neo-intimal SMCs and adventitial cells encapsulating the cultured vein rings differentially express versican

**Fig 5:**
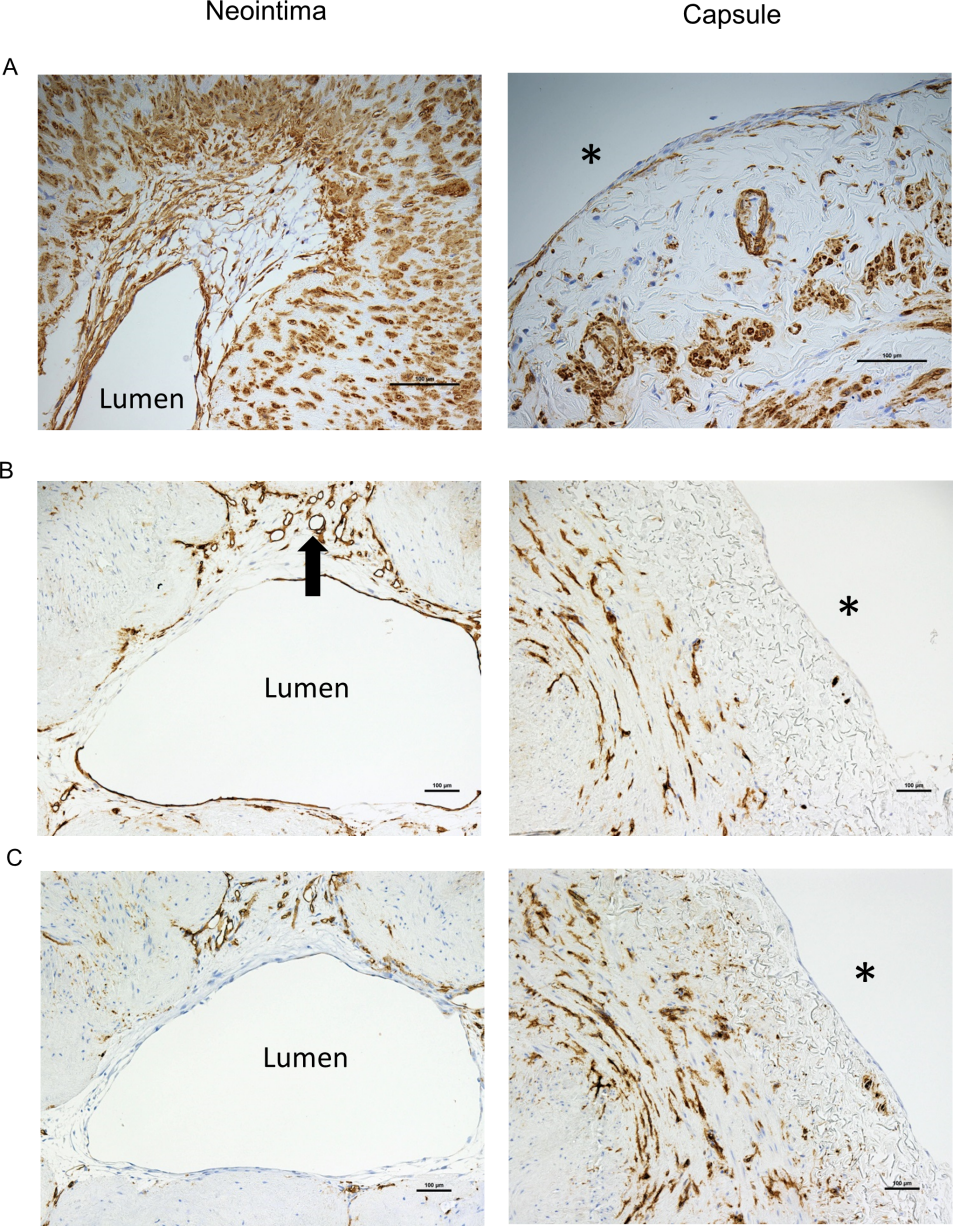
Immunostaining of cells in the neointima and adventitial capsule. Immunostaining of cells in the neointima and capsule surrounding the adventitia (left and right panels, respectively, for (A) smooth muscle alpha actin, (B) CD31 (arrow indicates an endothelialized neovessel), and (C) CD34. * indicates the adventitial surface. Scale bars indicate 100 μm.

**Fig 6:**
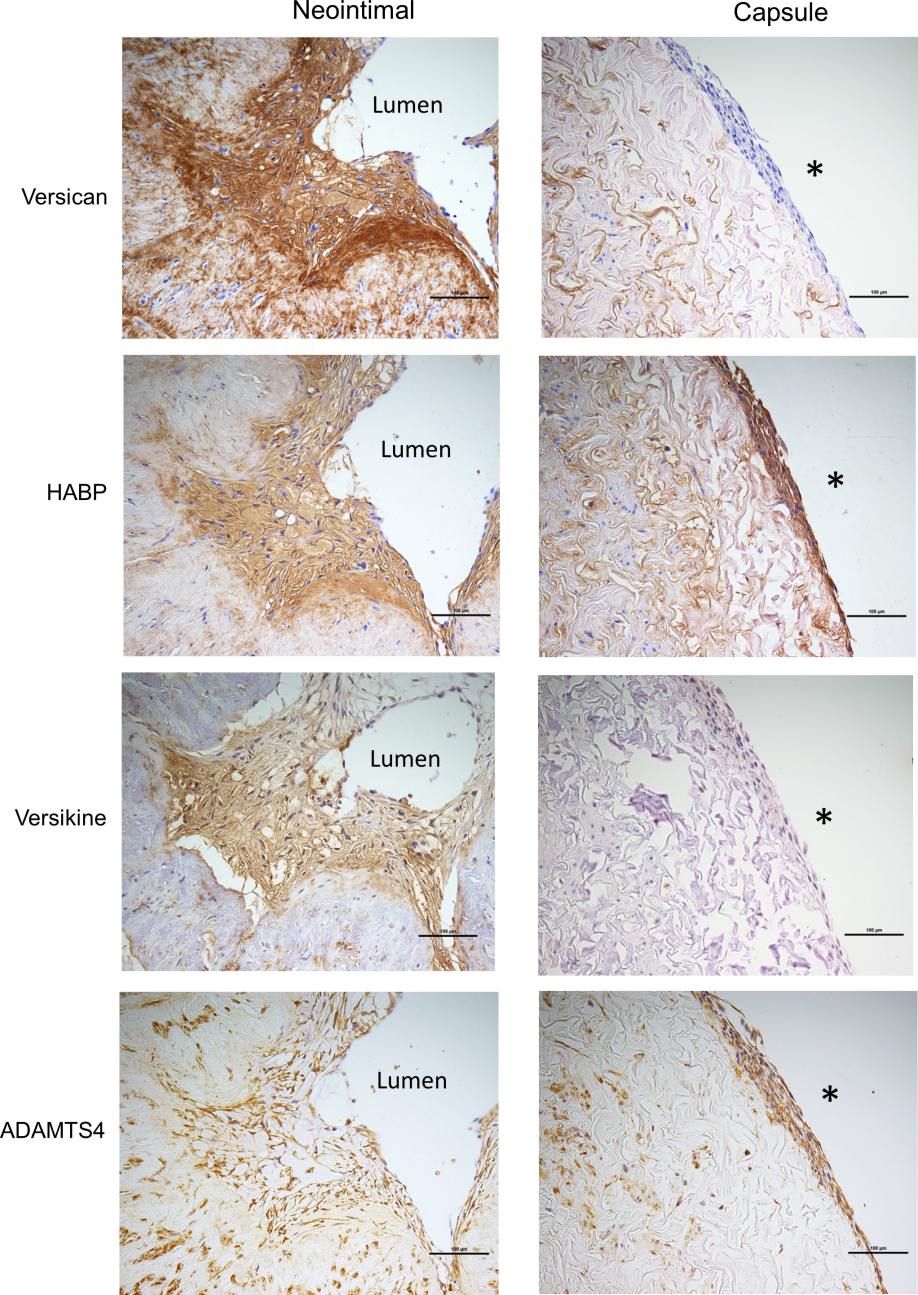
Immunostaining of cells in the neointima and adventitial capsule. Histochemical and immunostaining of neointimal cells and capsule cells (left and right panel, respectively) for (A) versican, (B) hyaluronan, (C) versikine, and (D) ADAMTS4. * indicates the adventitial surface. Scale bars indicate 100 μm.

After 14 days of vein ring culture, there were usually, but not always, neointimal cells observed lining the lumen of the vein rings, and a new cellular layer encapsulating the outer adventitial surface of the vein ring. While the neointimal cells were predominately positive for SMA, the encapsulating perimeter cells were not (Fig 5; 84 ± 5% and 28 ± 6% for paired neointimal and perimeter cells, respectively; N = 20 veins). We found that versican and versikine staining was stronger in neointimal cells compared to the adventitial perimeter cells (Fig 6), but both cells demonstrated strong HABP staining (Fig 6). ADAMTS4 immunostaining was stronger for the perimeter cells, while ADAMTS5 showed little staining for both neointimal and perimeter cells (the latter not shown). Finally, the perimeter cells were uniformly CD34/CD31 negative, while the only CD34- or CD31-positive neointimal cells were double positive endothelial cells observed in neointimal tubular structures (Fig 5; non-immune IgG had no signal [S1 Fig]). In conclusion, the cells encapsulating the adventitia are substantially different than the SMCs of the neointima.

### Versican and hyaluronan co-localize with the CD34^+^ progenitor cells in the vasa vasorum

**Fig 7:**
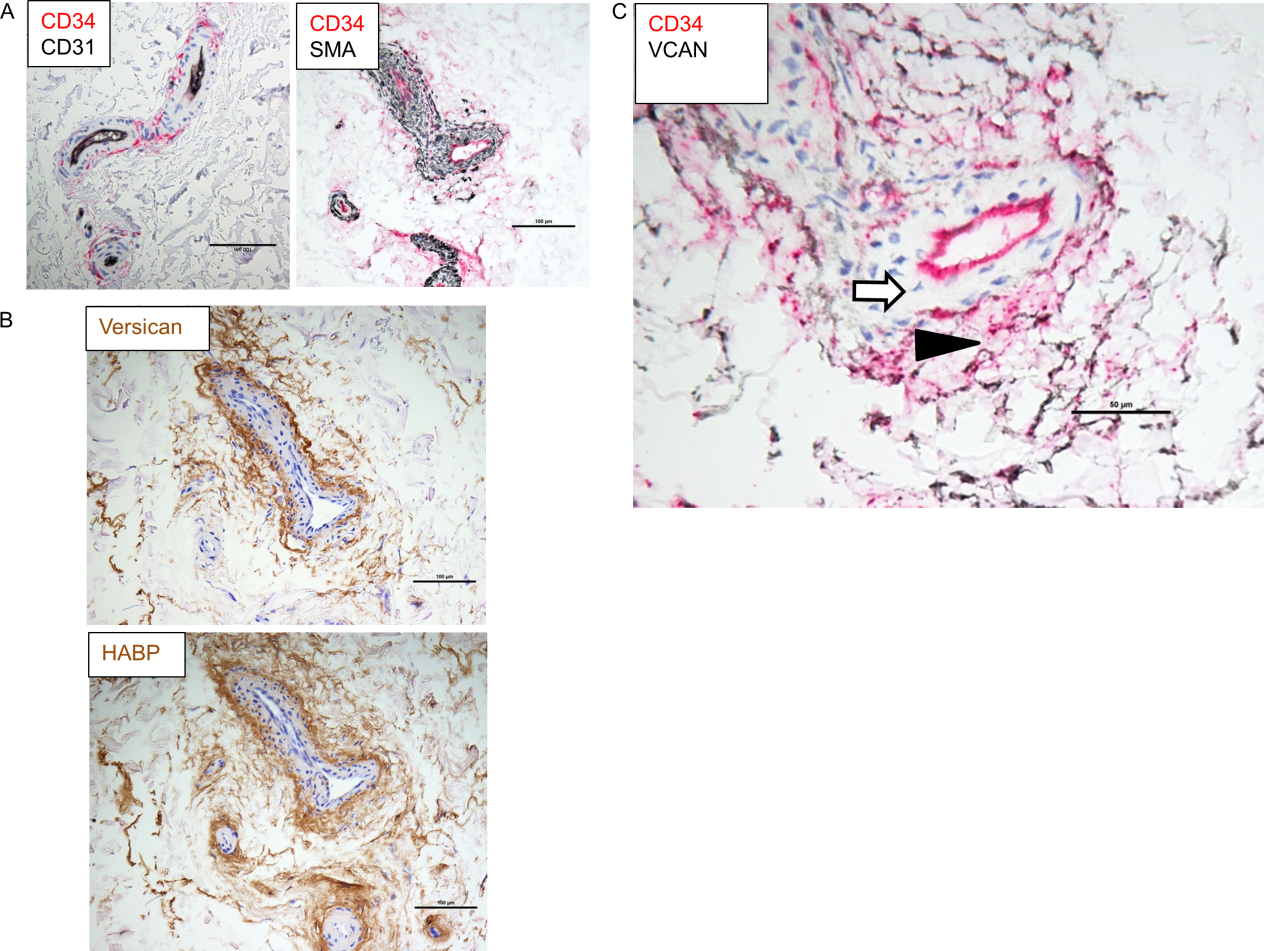
Immunostaining of the adventitial vasa vasorum. (A) The left panel shows an adventitial vessel in cross and tangential section stained for CD31 (black: endothelium) and CD34 (red: CD34^+^ progenitor cell and endothelium but the latter is masked by the black stain) and the right panel shows a cross and tangential section of a vessel stained for CD34 (red: CD34^+^ progenitor cell and endothelium) and SMA (black: pericytes). (B) Vessels stained for versican and hyaluronan (brown) in the top and bottom panels, respectively; and (C) a vessel stained for CD34 (red) and versican (black). The arrowhead indicates CD34 and versican co-localization compared to the arrow that indicates unlabeled pericytes. Scale bars indicate 100 μm in A and B, and 50 μm in C.

Versican and hyaluronan staining in the vasa vasorum showed distinct cell specificity. CD34^+^/CD31^-^ adventitial progenitor cells surround the SMA^+^ pericytes of small vessels (Fig 7A) [28]. Both versican and hyaluronan tended to be localized to the CD34^+^ adventitial progenitor cell layer, not to the pericytes or endothelial cells (Fig 7B). Double staining for versican and CD34 confirmed this co-localization (Fig 7C; black and red staining, respectively).

### Arterial pressure prevents induction of versican in vein grafts perfused ex vivo

**Fig 8:**
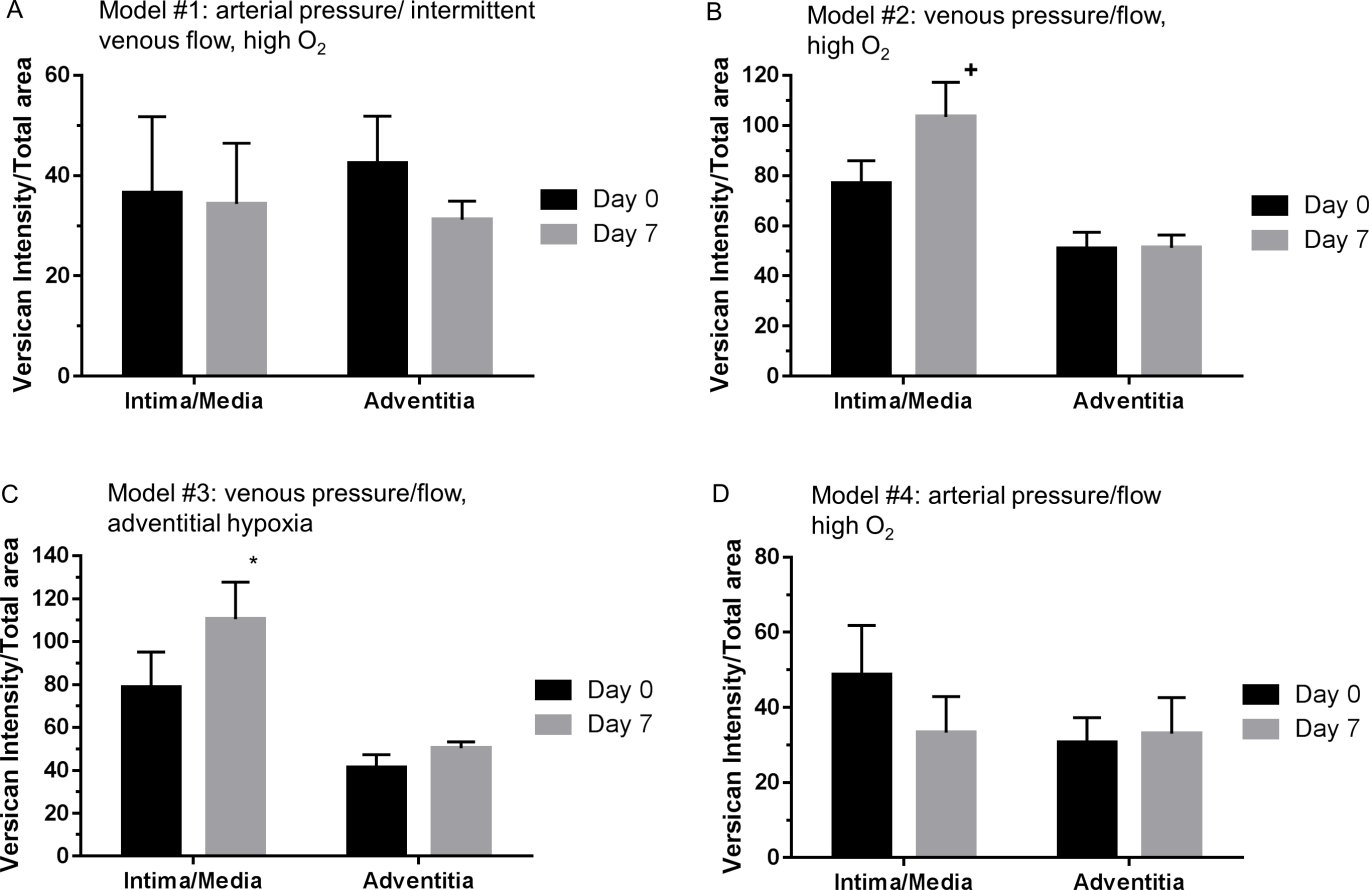
Versican expression levels in pre-culture and 7 day ex-vivo perfused veins. (A) Model #1: arterial pressure/ intermittent venous flow, high O_2_. (B) Model #2: venous pressure/flow, high O_2_. (C) Model #3: venous pressure/flow, adventitial hypoxia (D) Model #4: arterial pressure/flow high O_2_. *P<0.05 vs day 0, ^+^P<0.07 vs day 0 (black bars are day 0 and gray bars are day 7). N = 4 veins for models 1 and 4 and N = 5 veins for models 2–3.

During ex vivo culture of vein rings for 14 days, we observed that versican is increased in both intima/media and adventitia. We therefore perfused vein grafts ex vivo to further quantify the level of versican in the presence of pressure and flow. Patient information is presented in S1 Table. We generated 4 four different ex vivo perfusion models to assess the relative effects of pressure, flow, and levels of oxygen. There was no change in versican in either the intima/media or the adventitia in the two models with arterial pulsatile pressure, one with intermittent low flow and the other with continuous high arterial flow (models 1 and 4, respectively; Fig 8A and 8D). In contrast, in both models with low venous pressure and flow (one with 20% O_2_ on both sides and one with 20% O_2_ on the luminal side and 5% O_2_ on the adventitial side, models 2 and 3, respectively), there was increased versican or a trend (model 2; P = 0.07) for increased versican in the intima/media, but no change in adventitial versican (Fig 8B and 8C). These observations are summarized in Table 4.

**Table 4:** Versican changes in different ex vivo vein graft models.

Model	Description	Intimal/Medial Versican	Adventitial Versican
Floating Rings	No pressure or flow; 20% 0_2_	Increased	Increased
Ex Vivo Perfused 1	Intermittent high pulsatile pressure, venous flow; 20% 0_2_	No change	No change
Ex Vivo Perfused 2	Venous pressure & flow; 20% 0_2_	Trend increased	No change
Ex Vivo Perfused 3	Venous pressure & flow; 5% adventitial and 20% lumenal 0_2_	Increased	No Change
Ex Vivo Perfused 4	Arterial pulsatile pressure & flow; 20% 0_2_	No change	No change

### Cultured adventitial cells produce more versican and hyaluronan than SMCs

**Fig 9:**
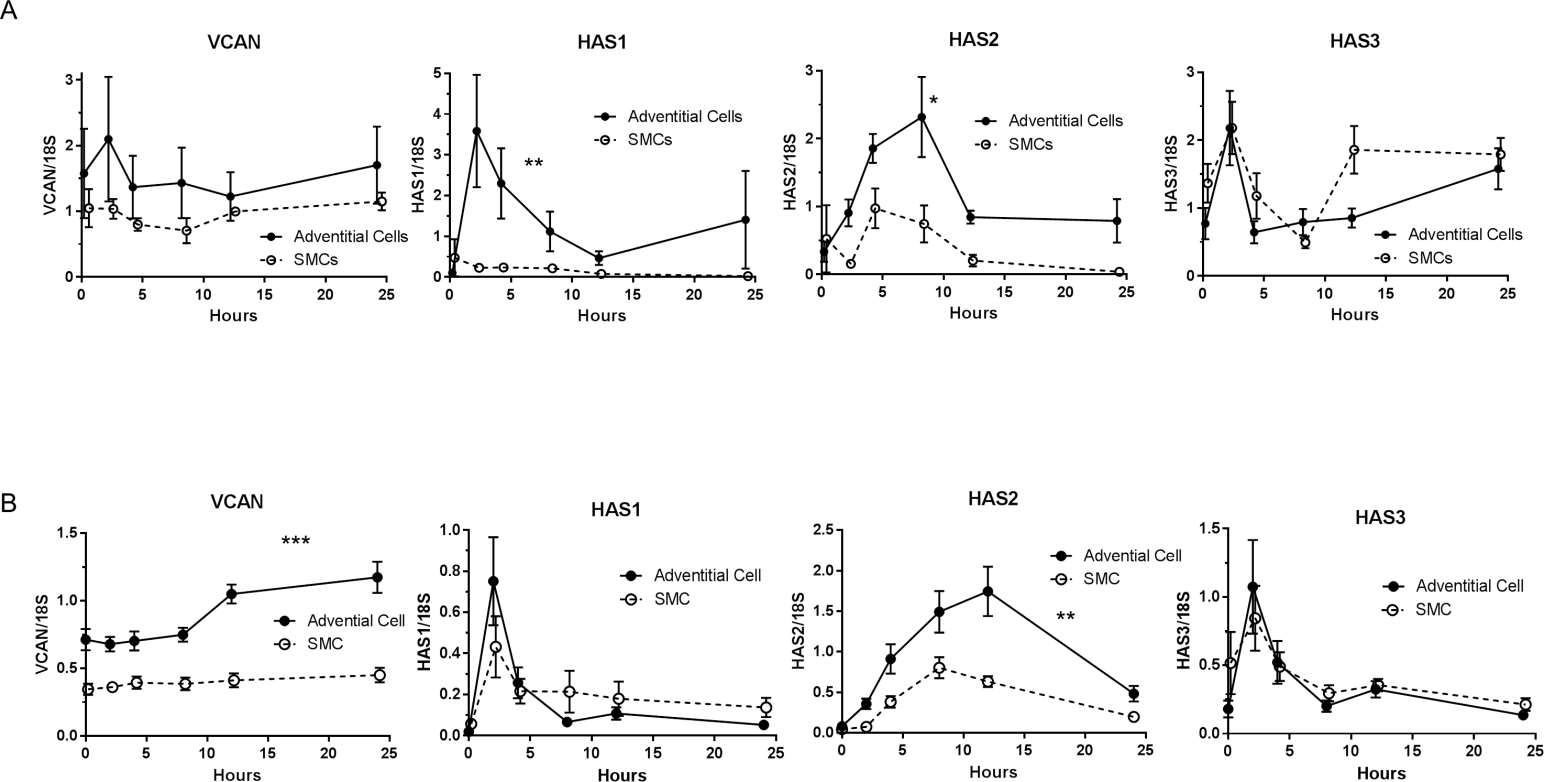
Induction of versican and HAS1-3 mRNA in adventitial cells and SMCs. Cells were stimulated with either 10% FBS (A) or 10 ng/ml PDGF-BB (B). Adventitial cells had significantly higher induction of versican, HAS1 and HAS2 compared to SMCs (***P<0.0001; **P<0.01; *P<0.05). Data are from pairs of cells from 3 (A) and 10 (B) veins in duplicate.

To further study differences between adventitia and intima/media, we compared the induction of versican and hyaluronan synthases in cultured venous SMCs and adventitial cells stimulated by FBS (as used in the tissue culture and ex vivo perfusion studies) or PDGF-BB, which has been shown to induce versican and hyaluronan production in arterial SMCs [29, 30]. We have previously shown that >90% of the SMCs are positive for SMA, while <10% of the adventitial cells are positive[10]. Neither of these cells express CD34[31]. Surprisingly, we did not observe an induction of versican in response to serum in either cell type, and HAS1 and HAS2 were induced in adventitial cells, but not SMCs (Fig 9A). HAS3 induction by serum was similar in the two cell types. In contrast to the responses to serum, PDGF-BB induced the expression of versican in adventitial cells, but not in SMCs (Fig 9B). HAS1 and HAS3 were induced to a similar extent in both cell types, but peak induction of HAS2 in adventitial cells was twice that of SMCs (Fig 9B). The amount of hyaluronan and versican protein in the cell layer and media of cultured adventitial cells was also higher than in SMCs (S3 Fig). Double immunofluorescent staining of the cells for versican and SMA demonstrated that there was no difference between the SMA negative and SMA positive cells, since all of the cells expressed some level of versican (S4 Fig).

## Discussion

In this study, we first documented the normal distribution of versican and hyaluronan in freshly resected human veins, and we further studied the changes in the distribution of these matrix proteins occurring in a variety of ex-vivo injury models that mimic the injury of surgical resection and reimplantation. The patients from whom these veins were obtained were receiving bypasses to address coronary and peripheral artery disease, and such patients commonly suffer from various co-morbidities [32]. Thus, it was not surprising to see the extent and variability of intimal hyperplasia present in these veins, which confirms prior studies [33, 34].

### Arterial pressure inhibits versican induction

The highest concentrations of versican were found in the intimal/medial layer, both in the fresh veins, and under the conditions of pressure-free, floating ring culture. These observations confirm the results of Merrilees et al.[35]. High pressure conditions suppressed the induction of versican (Table 3), compared to low pressure perfusion or no pressure (floating rings). It was further demonstrated that under high pressure, human saphenous vein SMCs in a tissue engineered blood vessel had less versican than normal human veins or arteries, regardless of pulsatile flow[36].

There are several possible explanations for this pressure effect, including the influences of miRNA, matrix metalloproteinases, and the effects of cell death. We have previously observed that miR138 is upregulated in model 1 (intermittent high pulsatile pressure with venous flow) and not in model 2 (venous pressure and flow) [14]. Since miR138 has been observed to bind the 3´ region of versican mRNA and decrease versican protein accumulation[37], the upregulation of miR138 may explain part of the reduced induction of versican under conditions of high pressure. In addition, TIMP1 is highly increased in the low flow, venous model (model 2) compared to the intermittent, high pressure model (model 1). TIMP1 would be expected to decrease the activity of many versican-degrading MMPs (e.g. MMP1, MMP2, MMP3, MMP7, and MMP9 [38–41]) in the static venous model. MMP2 and MMP9 are increased in both the static, venous (model 2) and static, high pressure models (model 1) [14]. These observations would be consistent with greater amounts of versican under low pressure because of less degradation by the MMPs. Disproportionate cell death in the intima/media might also explain the retarded accumulation of versican under pressure. Our own findings make this unlikely. Although there is a loss of medial SMCs in the high pressure models 1 and model 4, which is consistent with more complex CABG-like flow and pressure stimulations[42], we have also observed a loss of adventitial cells and SMCs in venous pressure/flow models (model 2) [12], and in the floating tissue model [43, 44].

Our current high pressure/high flow model results in a significant amount of neointimal hyperplasia (model 4), but does not show increased versican in the neointima. In contrast, studies of arterial injury in rats and pigs show increased versican protein in the arterial neointima at 7–8 days [45, 46]. However, there are little published data on the kinetics of versican in vein graft models. In rabbit vein grafts, Movat’s staining of proteoglycans and glycosaminoglycans, including versican and hyaluronan, increases over a period of weeks to months[47], while message levels for versican increase early and drop to baseline by 7–14 days (Dr. Scott Berceli, personal communication). Increased versican was observed after 2 weeks in porcine vein grafts [46]. Thus, it is possible that there is accumulation of versican in vein grafts perfused in vivo or ex vivo under pressure, but it occurs later than one week.

### Cultured adventitial cells show higher levels of versican and hyaluronan compared to cultured SMCs

We have shown that cultured human venous adventitial cells induce higher levels of versican and hyaluronan expression than SMCs. This was unexpected, given that at the tissue level, the venous medial tissue showed higher levels of versican and the same levels of hyaluronan compared to the adventitial tissue. Depending on the specific stimulus (i.e., PDGF vs FBS), HAS1, HAS2, and versican were induced more in adventitial cells than in SMCs. Unlike the current study, van den Boom et al. reported that PDGF did not induce HAS1 or HAS3 in human saphenous vein SMCs[48], however they looked only after 3 h using semi-quantitative qPCR and we observed peak activity for both after 2 h of treatment.

The mechanism for the increased versican induction in adventitial cells vs. SMCs is not clear. A simple explanation would be higher expression of PDGF receptors (PDGFRα and PDGFRβ) by adventitial cells, but we found no difference in expression of mRNA for these receptors between the two cell types[10]. Another possibility could be differences in the regulation of microRNAs[49]. For example, myocardin, which regulates expression of common SMC markers[50] and is expressed 11-fold higher in SMCs compared to adventitial cells (data not presented), also induces miR143. Interestingly, miR143 downregulates versican mRNA [51]. Finally, the long non-coding RNA, HAS2-AS1, has a pivotal role in the regulation of HAS2 transcription in vascular cells [52], however we previously observed no difference in the level of expression of HAS2-AS1 between the venous cell-types[31].

### Adventitial CD34^+^ progenitor cells and versican

Adventitial-cell specific induction of ECM genes is of particular interest because data from animal models supports a role for adventitial cells in intimal hyperplasia of engrafted veins[53–55] and because of the large amount of glycosaminoglycans, largely versican, present in failed vein graft intima[4]. In addition, a role for PDGF is relevant given that PDGF mediates cell migration in the human saphenous vein ex vivo[56] and given the large body of literature demonstrating a role for PDGF in the vascular response to injury[57]. In pig and rodent vein graft models 10–30% of intimal cells are derived from the adventitia. However, because of the limitations of human studies, the particular venous cells involved in human vein graft failure remain undefined. The venous adventitia contains many types of cells including endothelial cells, pericytes, CD34^+^ progenitor cells[28], longitudinal bundles of SMCs[58], macrophages, mast cells[59], and nerve cells[60]. We have previously observed that our cultured adventitial cells are >90% negative for SMA, in contrast to SMCs that are >90% positive[10]. This suggests the possibility that CD34^+^ progenitor cells, which are also SMA negative [28], are the precursors of our cultured adventitial cell rather than SMCs from longitudinal cords in the adventitia or pericytes of the vasa vasorum, which are both SMA positive. While the cultured adventitial cells do not express CD34[31], it is known that cultured CD34^+^ progenitor cells lose CD34 expression [28]. Moreover, the phenotypic plasticity of vascular cells is increasingly appreciated. It was recently reported that SMC-derived adventitial Sca1^+^ progenitor cells can differentiate in vivo into mature SMCs, resident macrophages, and endothelial-like cells[61]. Thus, identification of the specific human adventitial cell that migrates from adventitial explants remains a goal of further studies, especially given recent results suggesting a novel protective role for these cells[31]. In addition, our observation that versican and hyaluronan associate with CD34^+^ progenitor cells, but not pericytes, raises the unanswered question of what role these matrix factors have regarding progenitor cell function.

### Roles for versican and hyaluronan after vascular injury

Versican and hyaluronan are major components of the provisional ECM synthesized after injury and these factors and their degradation products (e.g. versikine) are well known to regulate vascular cell function [5, 6, 25]. For example, versican promotes SMC migration and proliferation[62]. In addition, HAS1 and HAS3 stimulate SMC migration[63, 64], and HAS2 stimulates SMC proliferation and migration[48][64]. The effects of hyaluronan are particularly complex as the size of the molecule, which is regulated by both the HAS isoform and hyaluronidase-mediated cleavage, dictates function with low molecular weight hyaluronan tending to be pro-inflammatory and high molecular weight hyaluronan tending to be anti-inflammatory[65, 66].

While we believe this is one of only a few studies to compare human saphenous vein adventitial cells with SMCs [10, 31, 67], there have been a number of investigations into differences between saphenous vein SMCs and arterial SMCs. These include greater rates of growth and migration and decreased rates of death for venous SMCs compared to arterial SMCs because of differences in the PTEN/PKB, RhoA, and p27^Kip1^ pathways[68–70]. There is also a greater production of proteoglycans, including versican, in cultured saphenous veins compared to arteries [35]. These studies are consistent with the greater propensity of vein bypass grafts to fail compared to arterial bypass grafts [71].

In summary, higher levels of versican are associated with venous intimal/medial SMCs than with adventitial cells in freshly isolated human veins. In cultured floating vein rings, the increase in intimal/medial versican results from increased synthesis, while increased adventitial versican results from the lack of gene induction plus decreased degradation. In ex vivo perfused veins, high pressure (as in an arterial bypass) inhibits the accumulation of versican. In contrast to the behavior of vein tissues, isolated cultured human venous adventitial cells produce more versican and hyaluronan than SMCs, which may participate in promoting migration and proliferation of the adventitial cells. Finally, we found that in the vasa vasorum versican and hyaluronan are associated with CD34^+^ progenitor cells rather than the SMC-like pericytes. These results demonstrate a differential regulation of versican and hyaluronan in adventitia vs intima/media, and raise the possibility of different functions for these ECM factors in adventitia compared to intima/media.

## Supporting information

S1 Fig Negative controls for antibodies and HABP. Examples of staining with antibodies to versican, versikine, ADAMTS4, ADAMTS5, CD31, and CD34 with paired non-immune IgGs are shown along with HABP with or without treatment with hyaluronidase. Scale bars are 500 μm for all except versikine, which is 100 μm.
(TIFF)

S2 Fig Examples of variability among veins for versican and hyaluronan. Staining of two veins for versican, HABP, and with Movat’s stain is shown. Scale bars are 500 μm.
(TIF)

S3 Fig Hyaluronan and versican in the cell layer and conditioned medium of cultured adventitial cells and SMCs. (A) Production of hyaluronan in adventitial cells and SMCs in response to 10 ng/ml PDGF-BB. Adventitial cells produce more hyaluronan than do SMCs (*p* < 0.01). *n* = 2 pairs of cells in duplicate. (B) Western blot of versican in the cell layer and (C) conditioned medium of adventitial cells compared to SMCs before and after 24 hours of treatment with PDGF-BB. The locations of the V0 and V1 isoforms of versican are indicated. AC = adventitial cell.
(TIF)

S4 Fig Double immunostaining of SMA and versican in cultured adventitial cells and SMCs. (A) Cells were treated for 24 hour with 10 ng/ml PDGF-BB before fixation and staining. (B) Quantification of SMA and versican positive cells from 3 pairs of adventitial cells and SMCs. * P<0.05.
(TIF)

S1 Table Patient Demographics in ex vivo vein graft models. (TIF)
