# Supplementary figures and images for: Versican is differentially regulated in the adventitial and medial layers of human vein grafts
Source: PLoS One. 2018 Sep 28;13(9):e0204045. doi: 10.1371/journal.pone.0204045 (PMC6161854; doi:10.1371/journal.pone.0204045)

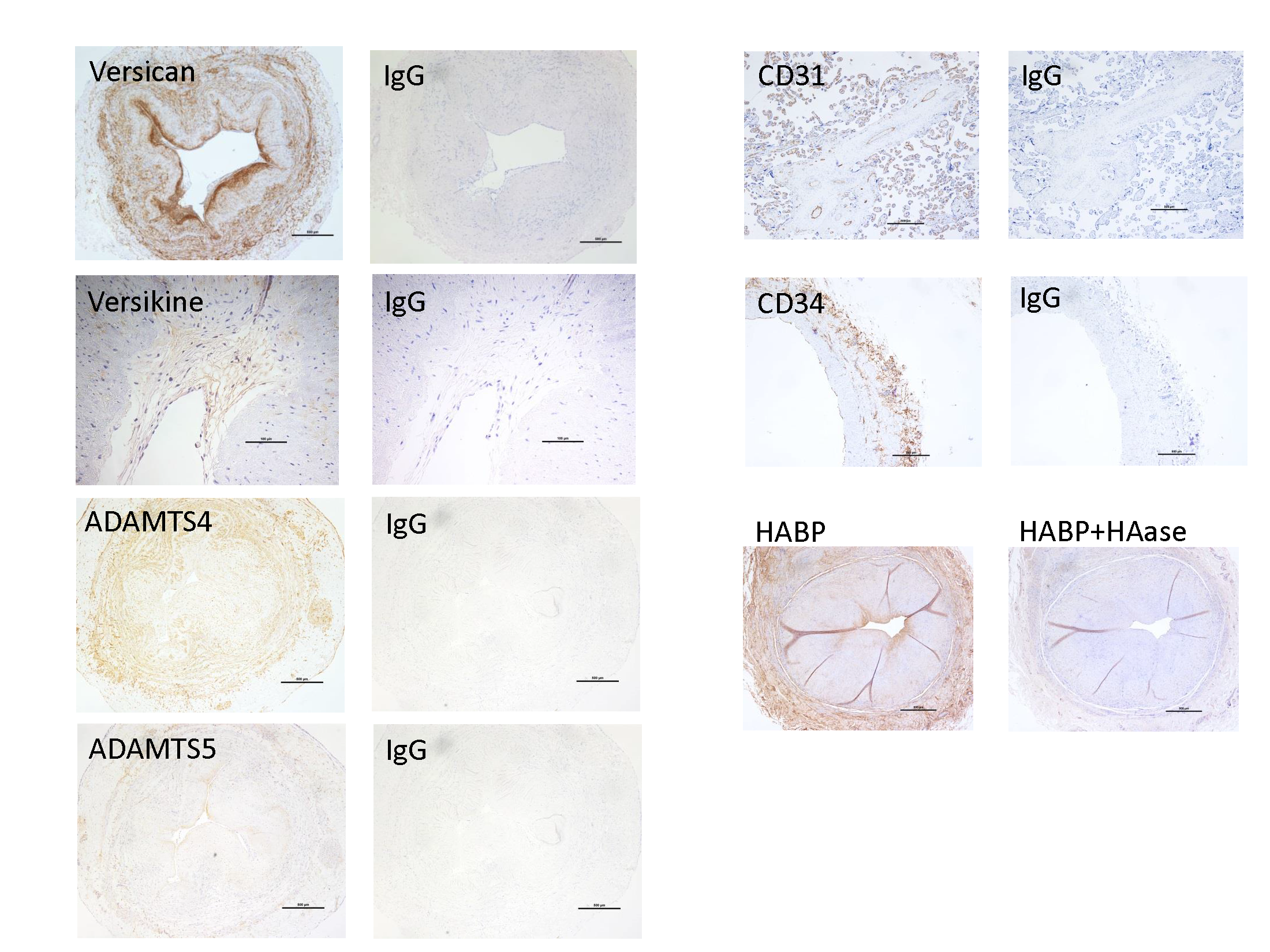

Supplement: S1 Fig — Examples of staining with antibodies to versican, versikine, ADAMTS4, ADAMTS5, CD31, and CD34 with paired non-immune IgGs are shown along with HABP with or without treatment with hyaluronidase. Scale bars are 500 μm for all except versikine, which is 100 μm. (TIFF) [file pone.0204045.s001.tiff]

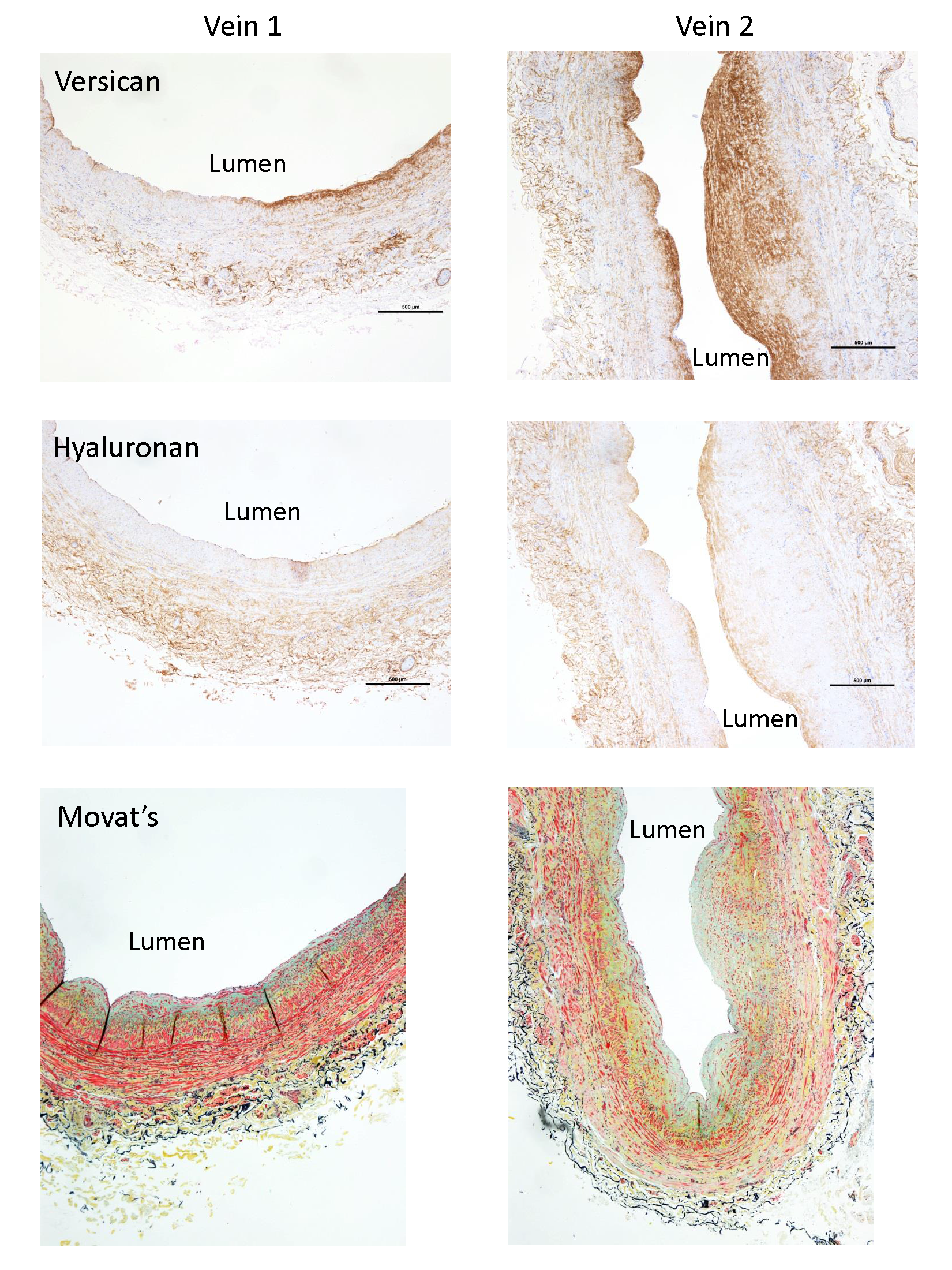

Supplement: S2 Fig — Staining of two veins for versican, HABP, and with Movat’s stain is shown. Scale bars are 500 μm. (TIF) [file pone.0204045.s002.tif]

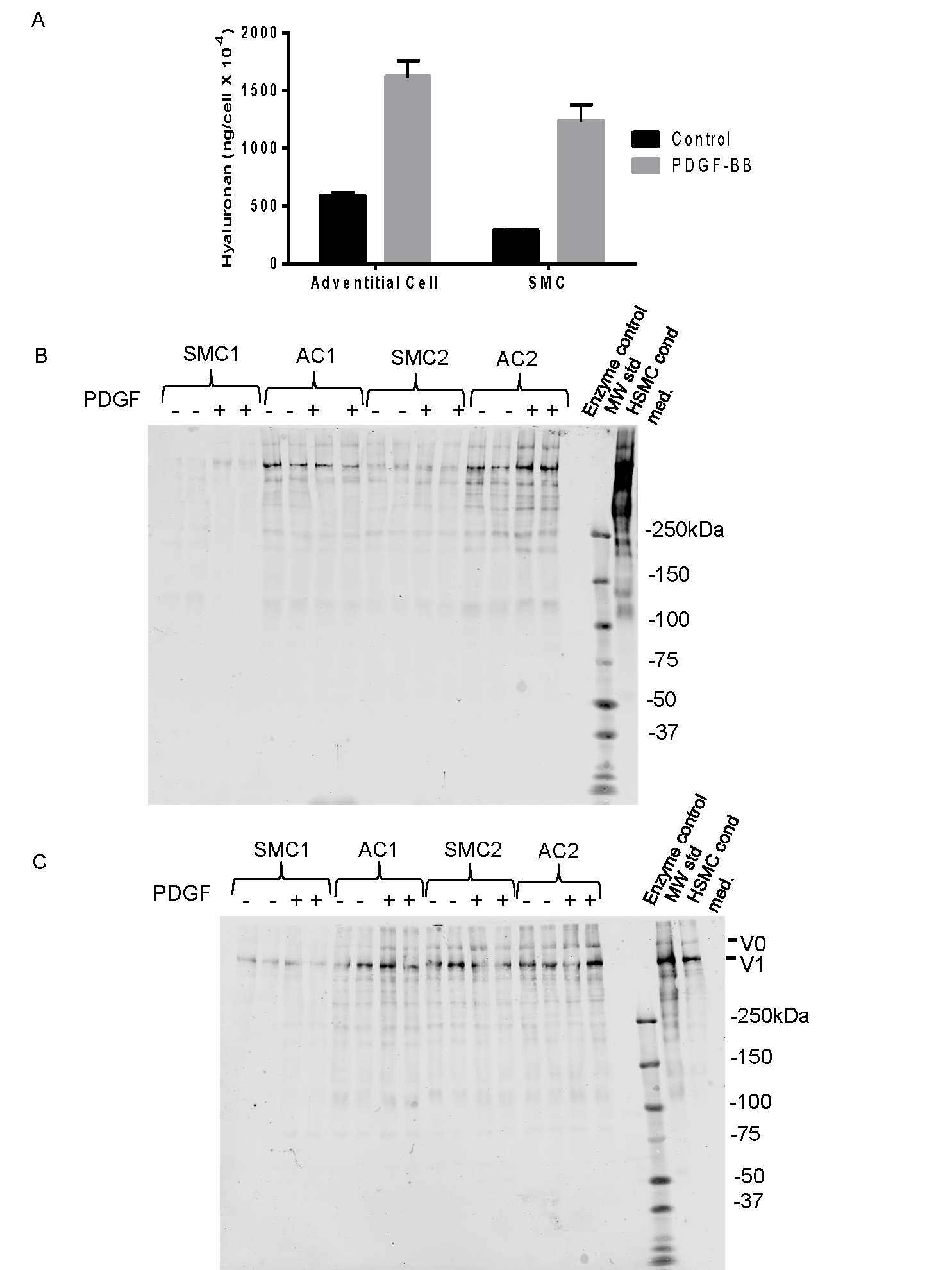

Supplement: S3 Fig — (A) Production of hyaluronan in adventitial cells and SMCs in response to 10 ng/ml PDGF-BB. Adventitial cells produce more hyaluronan than do SMCs (p < 0.01). n = 2 pairs of cells in duplicate. (B) Western blot of versican in the cell layer and (C) conditioned medium of adventitial cells compared to SMCs before and after 24 hours of treatment with PDGF-BB. The locations of the V0 and V1 isoforms of versican are indicated. AC = adventitial cell. (TIF) [file pone.0204045.s003.tif]

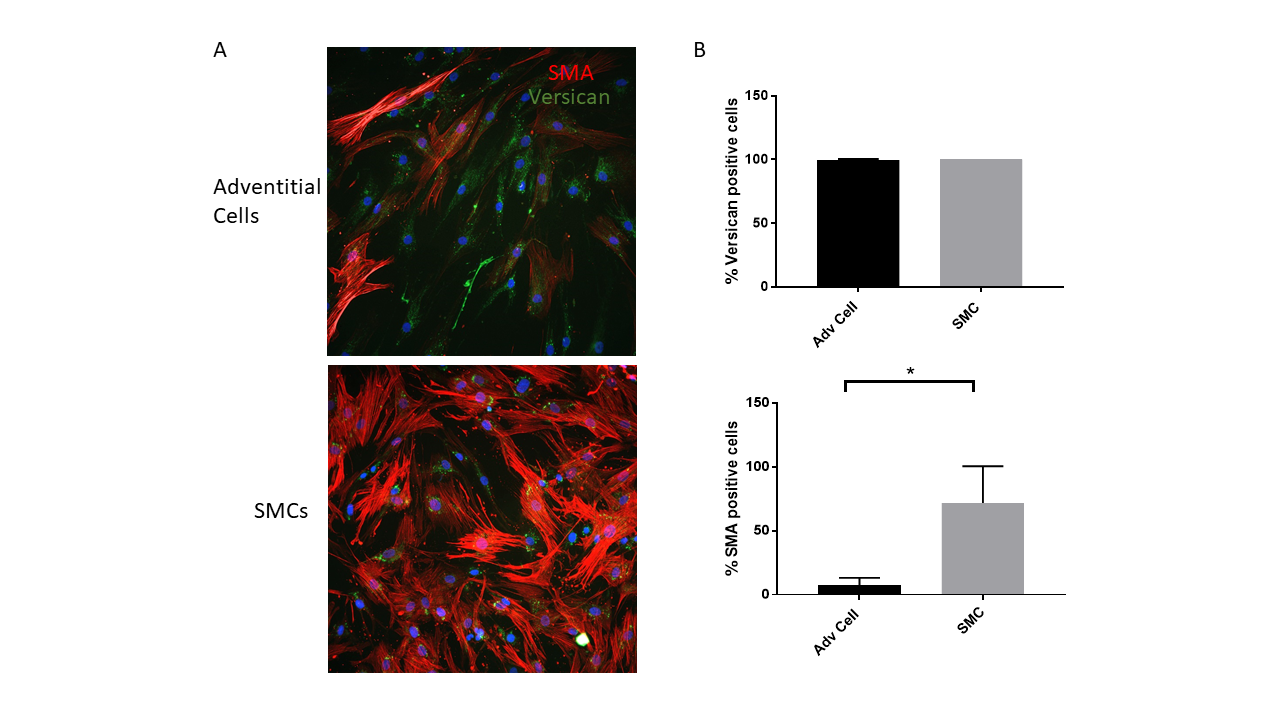

Supplement: S4 Fig — (A) Cells were treated for 24 hour with 10 ng/ml PDGF-BB before fixation and staining. (B) Quantification of SMA and versican positive cells from 3 pairs of adventitial cells and SMCs. * P<0.05. (TIF) [file pone.0204045.s004.tif]

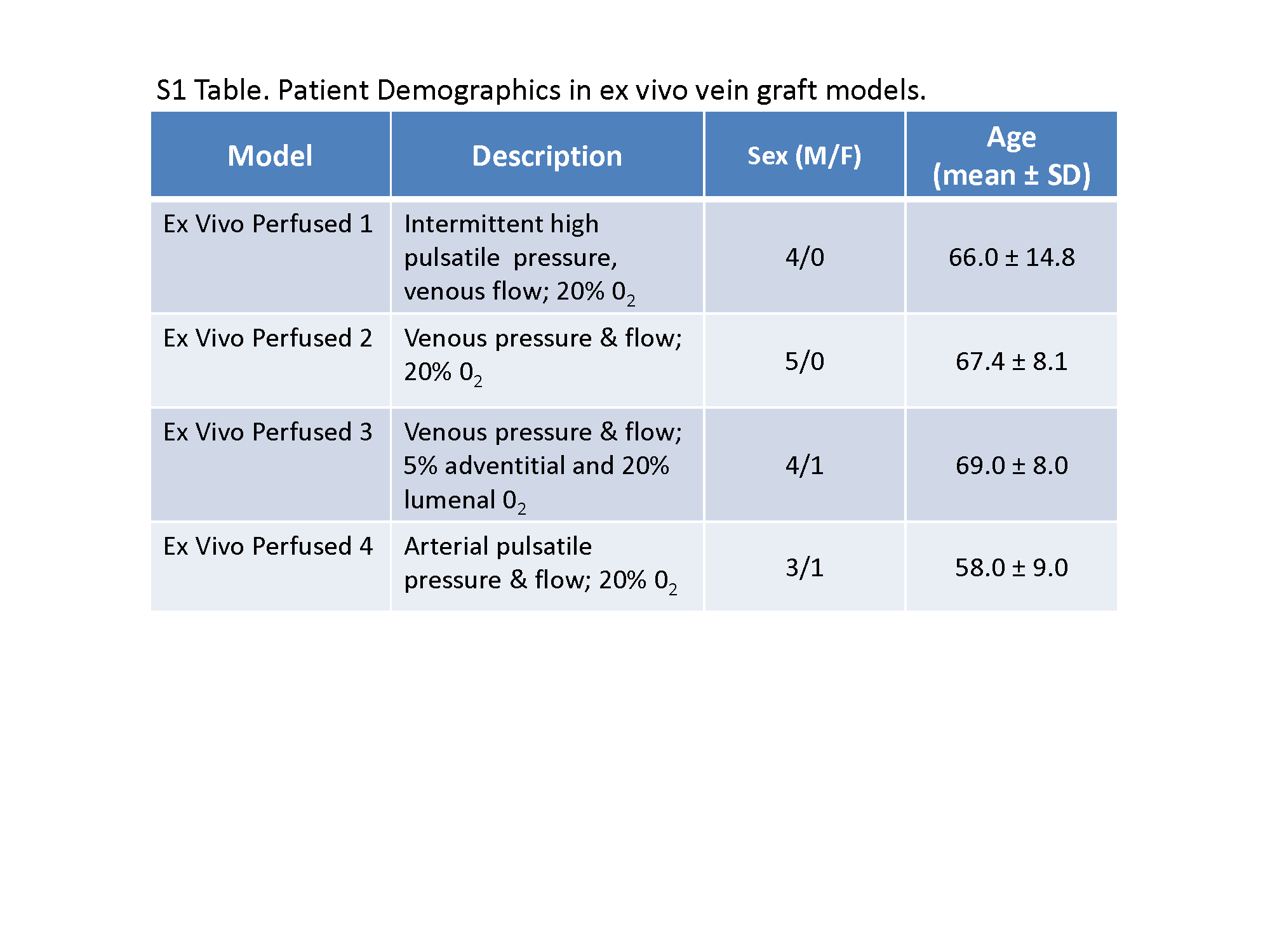

Supplement: S1 Table — (TIF) [file pone.0204045.s005.tif]
